# Reduction of VLDL Secretion Decreases Cholesterol Excretion in Niemann-Pick C1-Like 1 Hepatic Transgenic Mice

**DOI:** 10.1371/journal.pone.0084418

**Published:** 2014-01-03

**Authors:** Stephanie M. Marshall, Kathryn L. Kelley, Matthew A. Davis, Martha D. Wilson, Allison L. McDaniel, Richard G. Lee, Rosanne M. Crooke, Mark J. Graham, Lawrence L. Rudel, J. Mark Brown, Ryan E. Temel

**Affiliations:** 1 Department of Pathology, Section on Lipid Sciences, Wake Forest University School of Medicine, Winston-Salem, North Carolina, United States of America; 2 Cardiovascular Group, Antisense Drug Discovery, Isis Pharmaceuticals, Carlsbad, California, United States of America; 3 Department of Cellular and Molecular Medicine, Cleveland Clinic Foundation – Lerner Research Institute, Cleveland, Ohio, United States of America; 4 Saha Cardiovascular Research Center, University of Kentucky, Lexington, Kentucky, United States of America; St. Joseph's Hospital and Medical Center, United States of America

## Abstract

An effective way to reduce LDL cholesterol, the primary risk factor of atherosclerotic cardiovascular disease, is to increase cholesterol excretion from the body. Our group and others have recently found that cholesterol excretion can be facilitated by both hepatobiliary and transintestinal pathways. However, the lipoprotein that moves cholesterol through the plasma to the small intestine for transintestinal cholesterol efflux (TICE) is unknown. To test the hypothesis that hepatic very low-density lipoproteins (VLDL) support TICE, antisense oligonucleotides (ASO) were used to knockdown hepatic expression of microsomal triglyceride transfer protein (MTP), which is necessary for VLDL assembly. While maintained on a high cholesterol diet, Niemann-Pick C1-like 1 hepatic transgenic (L1Tg) mice, which predominantly excrete cholesterol via TICE, and wild type (WT) littermates were treated with control ASO or MTP ASO. In both WT and L1Tg mice, MTP ASO decreased VLDL triglyceride (TG) and cholesterol secretion. Regardless of treatment, L1Tg mice had reduced biliary cholesterol compared to WT mice. However, only L1Tg mice treated with MTP ASO had reduced fecal cholesterol excretion. Based upon these findings, we conclude that VLDL or a byproduct such as LDL can move cholesterol from the liver to the small intestine for TICE.

## Introduction

Atherosclerotic coronary vascular disease (ASCVD) remains the number one killer of Americans [1]. There is a strong positive relationship between low-density lipoprotein cholesterol concentration (LDLc) and ASCVD risk. One way to reduce LDLc, the primary risk factor of ASCVD, is to increase cholesterol excretion from the body [2]. Biliary cholesterol secretion is the primary mechanism by which excess cholesterol is moved into the lumen of the small intestine and subsequently excreted from the body [3]. However, our group and others have found that cholesterol excretion can also be facilitated by a non-biliary pathway known as transintestinal cholesterol efflux (TICE) [4], [5]. TICE appears to involve the movement of cholesterol through the plasma to the basolateral surface of the enterocytes. The cholesterol is then internalized, trafficked across the cell, and secreted into the lumen of the small intestine [4]. Under normal conditions in mice, TICE has been reported to contribute 20–50% of the cholesterol found in feces [6]–[8]. TICE has also been observed in humans but its quantitative contribution to fecal cholesterol excretion has not been established [9]. Activation of the nuclear hormone receptors liver X receptor (LXR) and peroxisome proliferator-activated receptor delta (PPARδ) with agonists has been shown to stimulate TICE in mice by ∼100% [6], [10]. In addition, when biliary cholesterol secretion is absent or dramatically reduced in mice and humans, normal cholesterol excretion can be maintained by TICE [7], [11]–[13]. For example, mice deficient in ABCB4 (ABCB4^−/−^) and mice with transgenic expression of Niemann-Pick C1-Like 1 in hepatocytes (L1Tg) have a ∼90% decrease in biliary cholesterol but normal fecal neutral sterol excretion presumably due to increased TICE [7], [8], [13]–[15]. It has also been shown that TICE can be pharmacologically stimulated with LXR agonist in ABCB4^−/−^ and L1Tg mice [7], [13], [14].

Lipoproteins must be involved in the trafficking of cholesterol to the enterocytes for TICE. Because of its importance in hepatobiliary cholesterol excretion [3], HDL is the lipoprotein class that most logically would support TICE. However, intestinal uptake of radiolabeled cholesteryl ether from HDL is unchanged in ABCB4^−/−^ mice, is increased in mice deficient in the HDL receptor scavenger receptor B-I (SR-BI), and is decreased in mice treated with LXR agonist [14]. In addition, TICE as measured by intestinal perfusion is increased in SR-BI deficient mice and unchanged in ATP binding cassette transporter A1 (ABCA1) deficient mice and mice deficient in both ABCA1 and SR-BI, which have extremely low levels of circulating HDL [12], [16]. Most recently, it was demonstrated that TICE does not depend on HDL-dependent delivery of plasma cholesterol to the intestine [16].

Based upon the evidence that HDL is not directly involved in TICE [7], [14], [17], we hypothesized that liver-derived, apolipoprotein B (apoB)-containing lipoproteins are delivering cholesterol to the small intestine for TICE. In the current study, we tested this hypothesis by selectively reducing the hepatic expression of microsomal triglyceride transfer protein (MTP), which is required for the assembly and secretion of apoB-containing very low density lipoproteins (VLDL) [18]. We found that inhibition of hepatic VLDL secretion reduced fecal neutral sterol excretion by >50% in L1Tg mice, a mouse model where TICE predominates. These studies suggest that an apoB-containing lipoprotein is responsible for moving cholesterol from the liver to the small intestine for TICE. We believe that our current findings will facilitate the discovery of other components of the TICE pathway and will open new avenues for the development of therapies that increase cholesterol excretion, lower LDLc, and reduce the incidence of ASCVD.

## Materials and Methods

### Mice

Male transgenic mice expressing human NPC1L1 in hepatocytes (L1Tg) [15] and wild type littermate controls on a C57BL/6N background were maintained on standard rodent chow. At 6 weeks of age, the mice were switched to a semisynthetic low-fat, high-cholesterol diet (10% of energy as palm-enriched fat, 0.2% cholesterol w/w) and were IP injected biweekly with 25 mg/kg of either non-targeting antisense oligonucleotide (Control ASO-ISIS 353512 (5′-TCCCATTTCAGGAGACCTGG-3′) [19] or ASO directed against murine MTP (MTP ASO-ISIS 144477 (5′-CCCAGCACCTGGTTTGCCGT-3′) as previously described [20]. After 6 weeks of treatment, mice were fasted for 4 hrs and anesthetized with ketamine/xylazine (120/20 mg/kg IM). Bile was collected from the gallbladder, and blood was collected by heart puncture for plasma isolation. Following a whole body flush with saline, liver and small intestine were collected and snap frozen in liquid nitrogen. All mice were maintained in an American Association for Accreditation of Laboratory Animal Care-approved animal facility under protocols approved by the institutional animal care and use committee at Wake Forest University School of Medicine.

### Immunoblotting of Tissue Proteins and Lipoprotein Apolipoproteins

Pieces of liver and proximal small intestine (<100 mg) were homogenized on ice in 25 mM Tris HCL pH 7.4, 300 mM NaCl and 1% Triton X-100 in the presence of protease inhibitor cocktail (Sigma). The tissue homogenate was centrifuged twice at 10,000 x *g* for 10 min and protein concentration of the supernatant was measured by Lowry assay [21]. Plasma pooled from 4–5 animals per group was separated by FPLC [22] and fractions corresponding to VLDL, LDL, transitional lipoprotein, and HDL were collected. After mixing with 5X SDS sample buffer, the tissue supernatant (10 µg protein/lane) and lipoprotein fractions were separated on Novex NuPAGE 4–12% Bis-Tris Midi Gels (Invitrogen). The proteins were transferred to nitrocellulose membranes, which were subsequently blocked with 5% (w/v) non-fat dried milk dissolved in wash buffer. The membranes were incubated with one or more of the following antibodies: mouse monoclonal to mouse MTP (BD Transduction Laboratories), rabbit monoclonal to LDL receptor (Abcam), rabbit polyclonal to ABCA1 (provided by Dr. John Parks, Wake Forest University School of Medicine), mouse monoclonal to β-actin (Sigma), goat polyclonal to human apoB (Academy Biomedical), rabbit polyclonal to rat apoE (provided by Dr. Joachim Herz, UT Southwestern Medical Center). After washing, the blots were probed with secondary antibodies against rabbit, goat, or mouse IgG conjugated to horseradish peroxidase (Sigma). Detected proteins were visualized with ECL reagent (PerkinElmer) and exposure to Blue X-Ray Film (Phenix).

### Quantitative Real-Time PCR (qPCR)

RNA extraction and qPCR was conducted as previously described on individual tissue samples (n = 5 per group) [23]. Cyclophilin was used as an internal control and mRNA expression levels were calculated based on the ΔΔ-CT method. Messenger RNA levels for each gene represent the amount relative to that of WT mice treated with control ASO, which was arbitrarily standardized to 1. Primer sequences used for qPCR are available on request.

### Plasma Concentration and Distribution of Cholesterol

Plasma total cholesterol concentration and lipoprotein cholesterol distributions were determined as described [22].

### In Vivo Determination of VLDL Lipid and ApoB Secretion Rates

After six weeks of high-cholesterol diet feeding and ASO treatment, mice (n = 5 per treatment group) were fasted for 4 hrs, anesthetized with isoflurane (4% induction & 2-3% maintenance, inhalation), and injected retro-orbitally with 1) [^35^S]Met/Cys (7 µCi/g body weight) to radiolabel newly synthesize apoB and 2) tyloxapol (500 mg/kg; Sigma) to block lipolysis [24]. At 0, 0.5, 1, 2, and 3 hrs after injection, 50 µl of blood was collected from anesthetized mice by retro-orbital bleeding. Plasma was harvested from the blood samples and used to quantify TG and total cholesterol (TC) mass by enzymatic assay. TG and TC secretion rates were derived from the slope of the line of best fit of time *versus* plasma TG and TC for each individual animal using GraphPad Prism 5. Hepatic secretion of newly synthesized apoB was measured in plasma from the 3 hr time point as described previously [25].

### Liver and Gallbladder Lipid Measurements

Lipid concentrations in liver and gallbladder bile were determined as described previously [15].

### Analysis of fecal neutral sterol excretion

After 6 weeks of treatment, fecal neutral sterol excretion was measured as described previously [23].

### Statistical Analysis

Data are expressed as the mean ± standard error of the mean (SEM), and were analyzed using multivariate analysis of variance (ANOVA) followed by Student's t tests for post hoc analysis. Differences were considered significant at p<0.05. All analyses were performed using JMP version 5.0.12 (SAS Institute; Cary, NC) software unless otherwise specified.

## Results

### MTP ASO treatment reduces hepatic MTP expression and function

**Figure 1:**
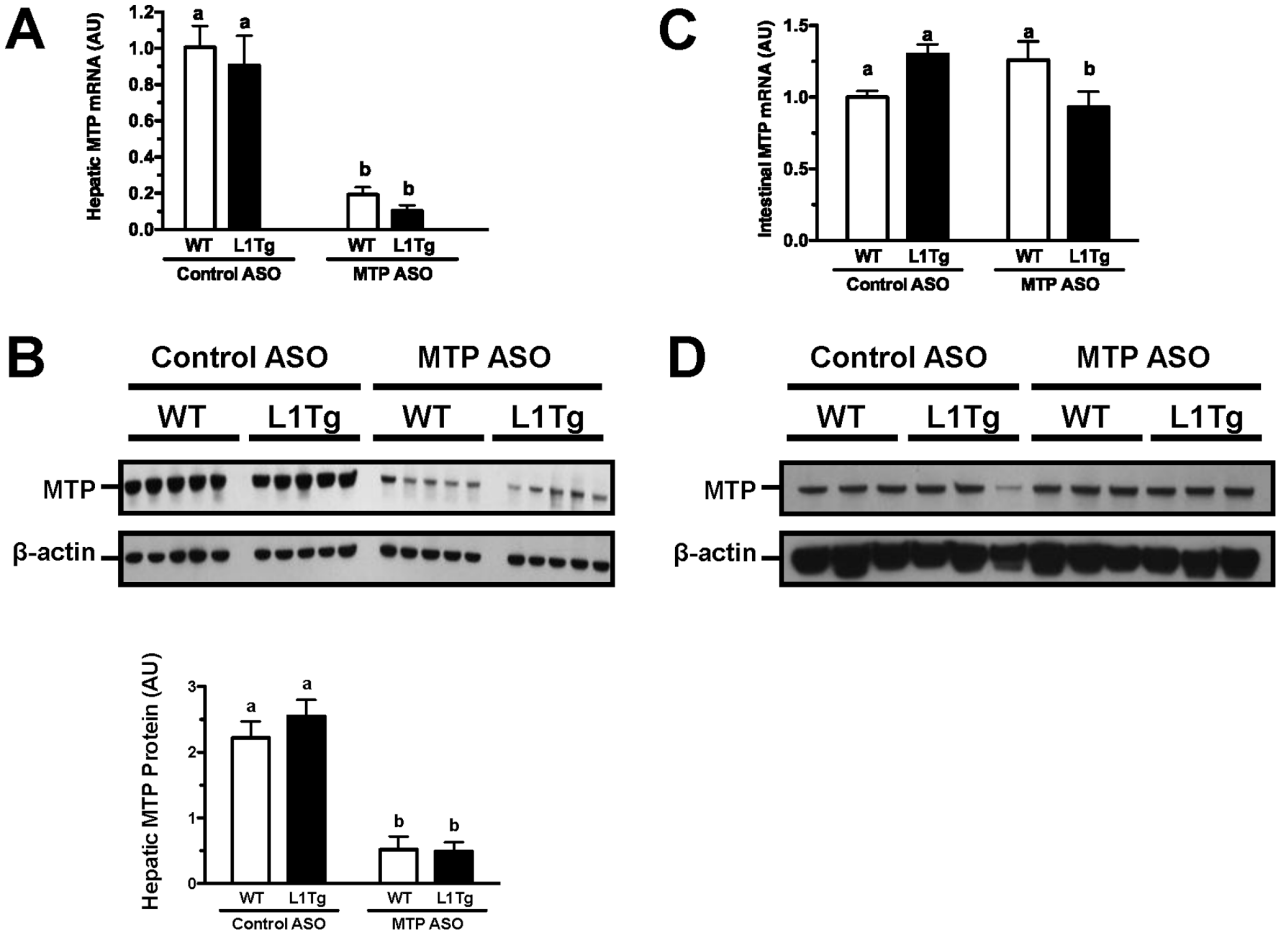
Hepatic and intestinal MTP expression following MTP ASO treatment. Liver and small intestine were collected from L1Tg and WT mice following 6 weeks of treatment with control ASO or MTP ASO. Quantitation of MTP mRNA in liver (A) and proximal small intestine (C) was conducted by real-time PCR using individual RNA samples (n = 5 per treatment group). Western blot analysis of MTP and β-actin in liver (B) and proximal small intestine (D). Data in graphs represent the means ± SEM, and means not sharing a common superscript differ significantly (p<0.05).

Male L1Tg and WT littermate controls mice were fed a low-fat, high-cholesterol diet and treated with either a non-targeting antisense oligonucleotide (control ASO) or an ASO targeting MTP (MTP ASO). Because ASOs are cleared more efficiently by the liver compared to the small intestine [26], we anticipated that knockdown of MTP expression would be significantly greater in liver versus small intestine, which requires MTP for assembly and secretion of chylomicrons [18]. After six weeks of treatment, hepatic MTP mRNA expression was reduced by ∼90% in both WT and L1Tg mice treated with MTP ASO compared to control ASO (Figure 1A). Hepatic MTP protein expression was reduced >75% with MTP ASO in both genotypes (Figure 1B). As expected, intestinal MTP mRNA (Figure 1C) and protein (Figure 1D) were minimally affected with MTP ASO treatment.

**Figure 2:**
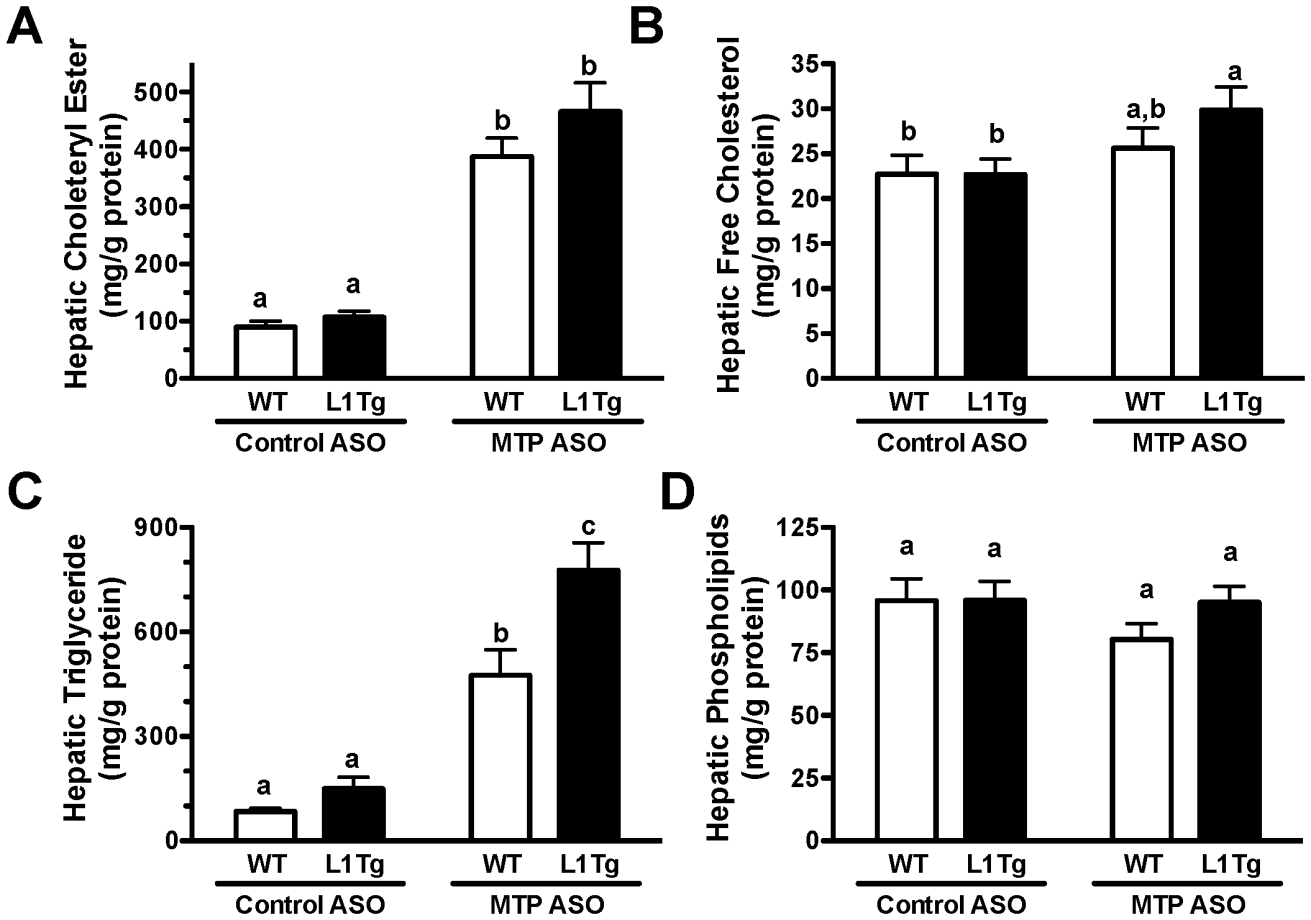
Liver lipid levels in mice with hepatic MTP knockdown. After 6 weeks of control ASO or MTP ASO treatment, fasting liver samples were collected and analyzed for the concentrations of cholesteryl ester (A), free cholesterol (B), triglyceride (C) and phospholipid (D). All hepatic lipid values were normalized to the protein content of the extracted tissue, and represent the means ± SEM (9–10 mice per treatment group). Means not sharing a common superscript differ significantly (p<0.05).

Since MTP is required for efficient efflux of hepatic lipids on apoB-containing lipoproteins, liver lipid accumulation was dramatically increased in mice treated with MTP ASO. Compared to WT mice treated with control ASO, WT and L1Tg mice treated with MTP ASO had a 330% and 420% increase, respectively, in hepatic cholesteryl ester (CE) (Figure 2A). MTP ASO treatment significantly increased hepatic free cholesterol (FC) content by 31% in L1Tg but not WT mice (Figure 2B). Liver triglyceride (TG) concentration was raised by 460% and 820% in WT and L1Tg mice respectively with hepatic MTP knockdown (MTP^HKD^) (Figure 2C). Hepatic phospholipid (PL) content was similar amongst treatment groups (Figure 2D).

### Hepatic MTP knockdown reduces fecal cholesterol excretion in L1Tg mice

**Figure 3:**
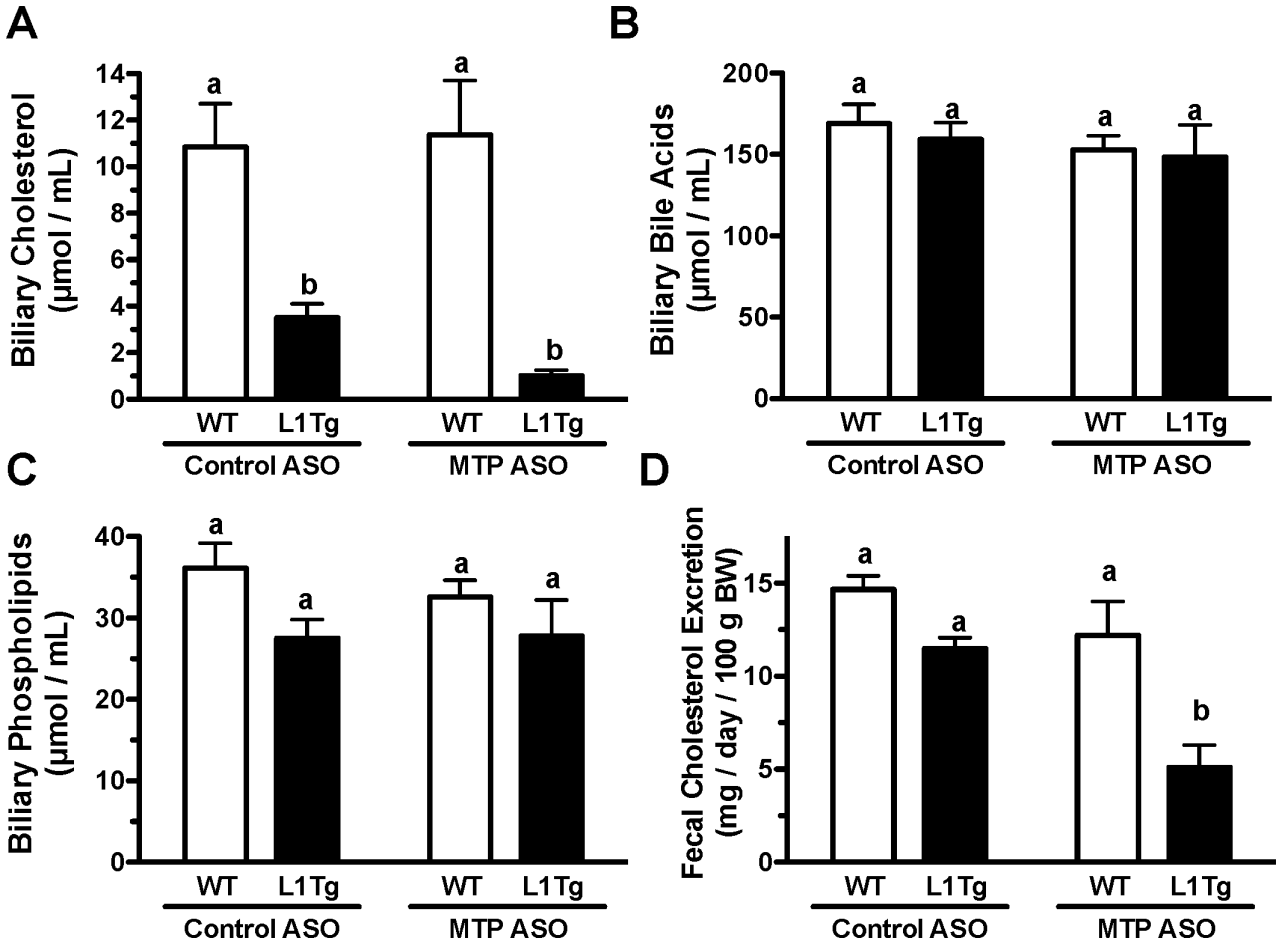
Biliary lipid levels and fecal cholesterol excretion in mice with hepatic MTP knockdown. After 6 weeks of control ASO or MTP ASO treatment, gallbladder bile was collected and analyzed for the concentration of cholesterol (A), bile acids (B), and phospholipids (C). For 3 days prior to euthanasia, feces were quantitatively collected for analysis of fecal cholesterol excretion (D). Data represent the means ± SEM (n = 7–10 per treatment group), and means not sharing a common superscript differ significantly (p<0.05).

Due to the increased cholesterol accumulation in liver (Figure 2A-B), MTP^HKD^ was expected to cause a compensatory elevation in biliary cholesterol concentration. Consistent with our previous work [13], [15], biliary cholesterol was reduced by 68% in L1Tg versus WT mice treated with control ASO. However, MTP ASO treatment did not increase biliary cholesterol in either WT or L1Tg mice (Figure 3A). The liver can also convert excess cholesterol to bile acids, but MTP^HKD^ had no effect on bile acid concentration in gallbladder bile (Figure 3B). The level of biliary phospholipids was also similar amongst the treatment groups (Figure 3C).

Despite the dramatic reduction in biliary cholesterol concentration (Figure 3A), control ASO-treated L1Tg mice compared to WT mice had normal fecal cholesterol excretion (Figure 3D), which has been attributed to increased TICE in L1Tg mice [13]. Fecal cholesterol excretion was unchanged in WT mice with MTP^HKD^ (Figure 3D). However, L1Tg mice treated with MTP ASO versus control ASO displayed a significant 53% reduction in fecal cholesterol loss (Figure 3D). These data indicate that inhibition of MTP-dependent hepatic lipid secretion on apoB-containing lipoproteins reduces TICE in L1Tg mice.

### Hepatic MTP knockdown decreases but does not eliminate hepatic secretion of apoB-containing lipoproteins

**Figure 4:**
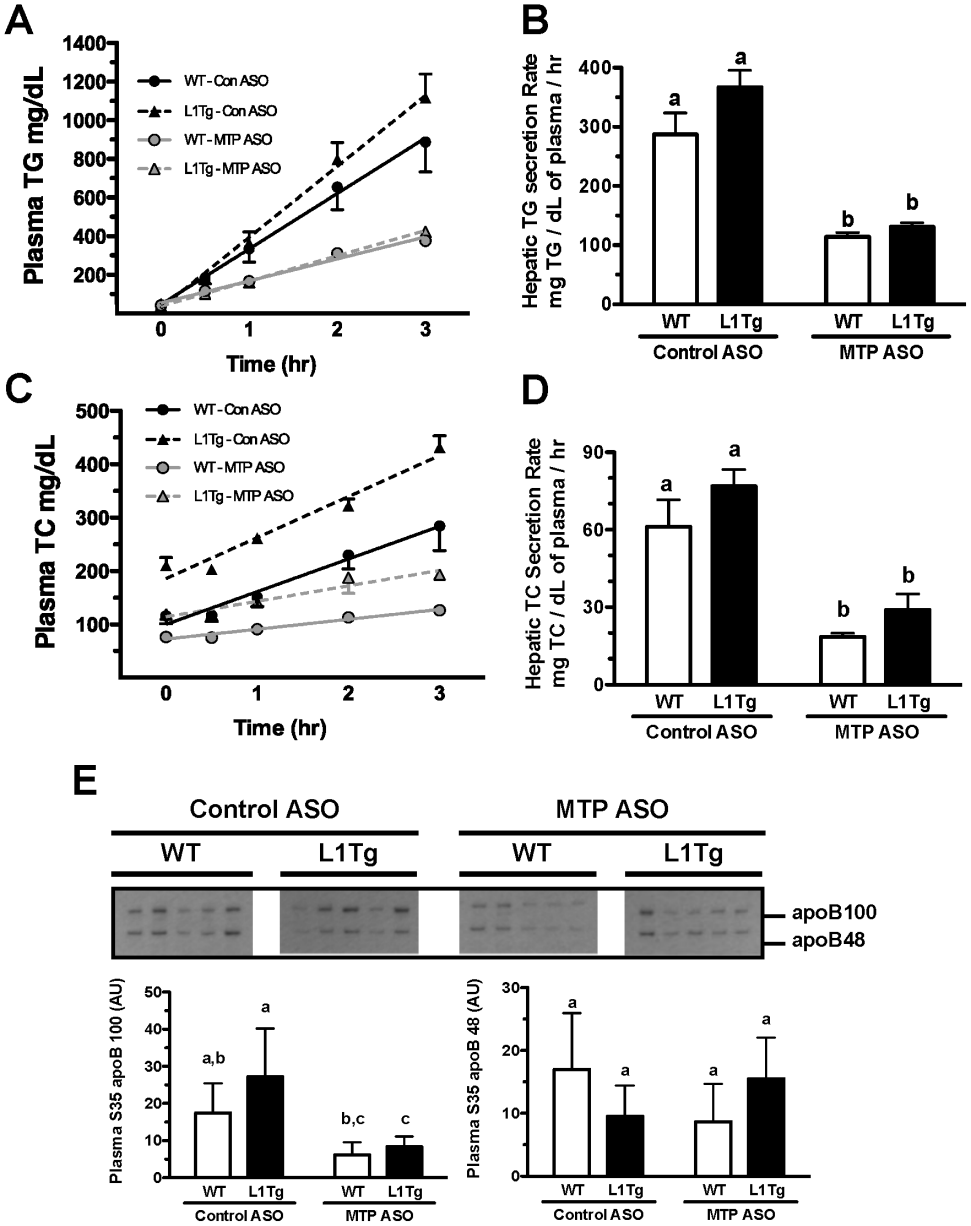
Hepatic secretion of lipid and apoB with MTP knockdown. Following 6 weeks of treatment with control ASO or MTP ASO, L1Tg and WT mice were fasted for 4-orbitally with tyloxapol (500 mg/kg) and [35S]Met/Cys. Blood samples were periodically collected and the plasma was analyzed for TG (A) and TC (C) concentration. The hepatic secretion rates of TG (B) and TC (D) were determined by linear regression analysis. Secretion of newly synthesized apoB100 and apoB48 (E) was measured by autoradiography of radiolabeled apoB that had been immunoprecipitated from plasma collected 3 hrs post-tyloxapol injection. The autoradiography data in panel E show samples that were separated on one gel and were exposed for the same time to the same piece of X ray film. Data represent the means ± SEM (n = 5 per treatment group), and means not sharing a common superscript differ significantly (*p*<0.05).

Since MTP ASO treatment reduced but did not abolish cholesterol excretion in L1Tg mice, the hepatic secretion of lipid and apoB was assessed in mice injected with tyloxapol and [^35^S]Met/Cys [24]. MTP^HKD^ in WT and L1Tg mice caused the hepatic secretion rate of TG (Figure 4A,B) to be decreased by ≥60% and total cholesterol (TC) (Figure 4C,D) to be reduced by >50%. MTP ASO versus control ASO treatment caused hepatic secretion of newly synthesized apoB100 to be significantly reduced in L1Tg mice and to trend towards a decrease in WT mice (Figure 4E). In contrast, hepatic apoB48 secretion was unchanged with MTP^HKD^ in WT and L1Tg mice (Figure 4E).

### Hepatic MTP knockdown reduces plasma VLDL but not LDL

**Figure 5:**
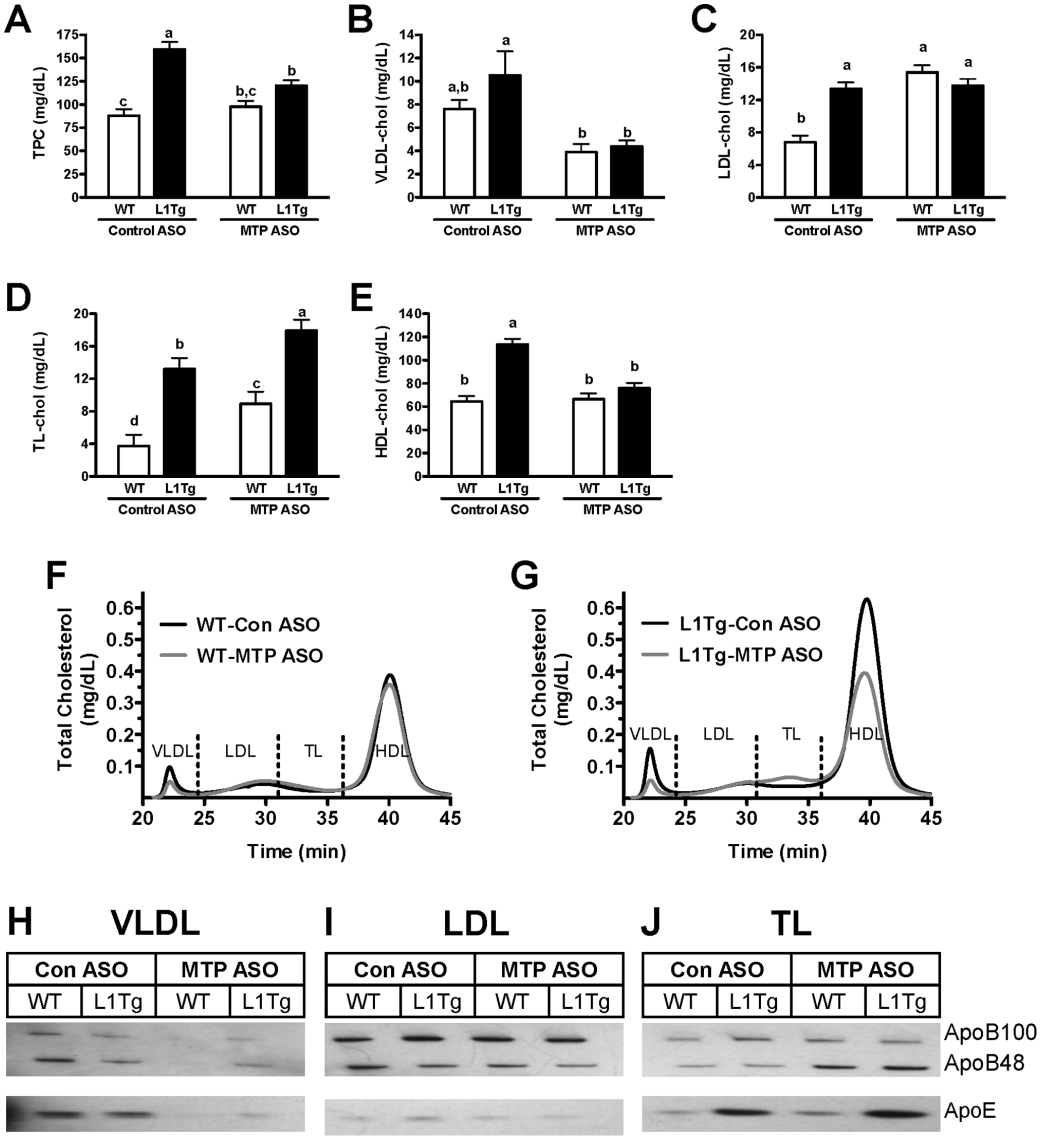
Plasma lipoprotein cholesterol and apoliporotein distribution following MTP knockdown. After treatment for 6 weeks with control ASO or MTP ASO, fasting plasma was collected from WT and L1Tg mice and analyzed for total cholesterol (A) and lipoprotein cholesterol distribution, which were used to calculate the cholesterol concentration in VLDL (B), LDL (C), transition lipoprotein [TL] (D) and HDL (E). Data represent the means ± SEM (n = 9–10 mice per treatment group), and means not sharing a common superscript differ significantly (*p*<0.05). An equal volume of pooled plasma from 4–5 mice per treatment group was separated by FPLC (F & G) and fractions containing VLDL (H), LDL (I), and TL (J) were collected. Following SDS-PAGE, the lipoprotein fractions were immunoblotted to determine the content of apoB and apoE.

To further evaluate the impact of hepatic MTP knockdown on apoB-containing lipoprotein levels, plasma lipoprotein cholesterol and apolipoprotein distribution was measured. Consistent with our previous study [15], plasma TC (Figure 5A) and HDL cholesterol (HDLc) (Figure 5E) were significantly increased in L1Tg versus WT mice treated with control ASO. In addition, control ASO-treated L1Tg mice had significantly more cholesterol associated with transition lipoproteins (TL) (Figure 5D), which were previously characterized as large, apoE-rich HDL [15]. As expected, MTP^HKD^ tended to reduce and significantly reduced VLDL cholesterol (VLDLc) in WT and L1Tg mice, respectively (Figure 5B). However, with MTP ASO treatment, LDL cholesterol was significantly increased in WT mice and was unchanged in L1Tg mice (Figure 5C), and TL cholesterol was significantly elevated in both genotypes (Figure 5D). MTP^HKD^ caused a significant reduction in HDLc in L1Tg mice but no change in HDLc in WT mice (Figure 5E). Consistent with the reduction in VLDLc, apoB100, apoB48 and apoE were reduced in VLDL from WT and L1Tg mice treated with MTP ASO (Figure 5H). In contrast, MTP^HKD^ did not affect apoB100, apoB48, and apoE levels in LDL (Figure 5I), and increased apoB48 in TL (Figure 5J).

### Hepatic MTP knockdown decreases LDL receptor in liver

**Figure 6:**
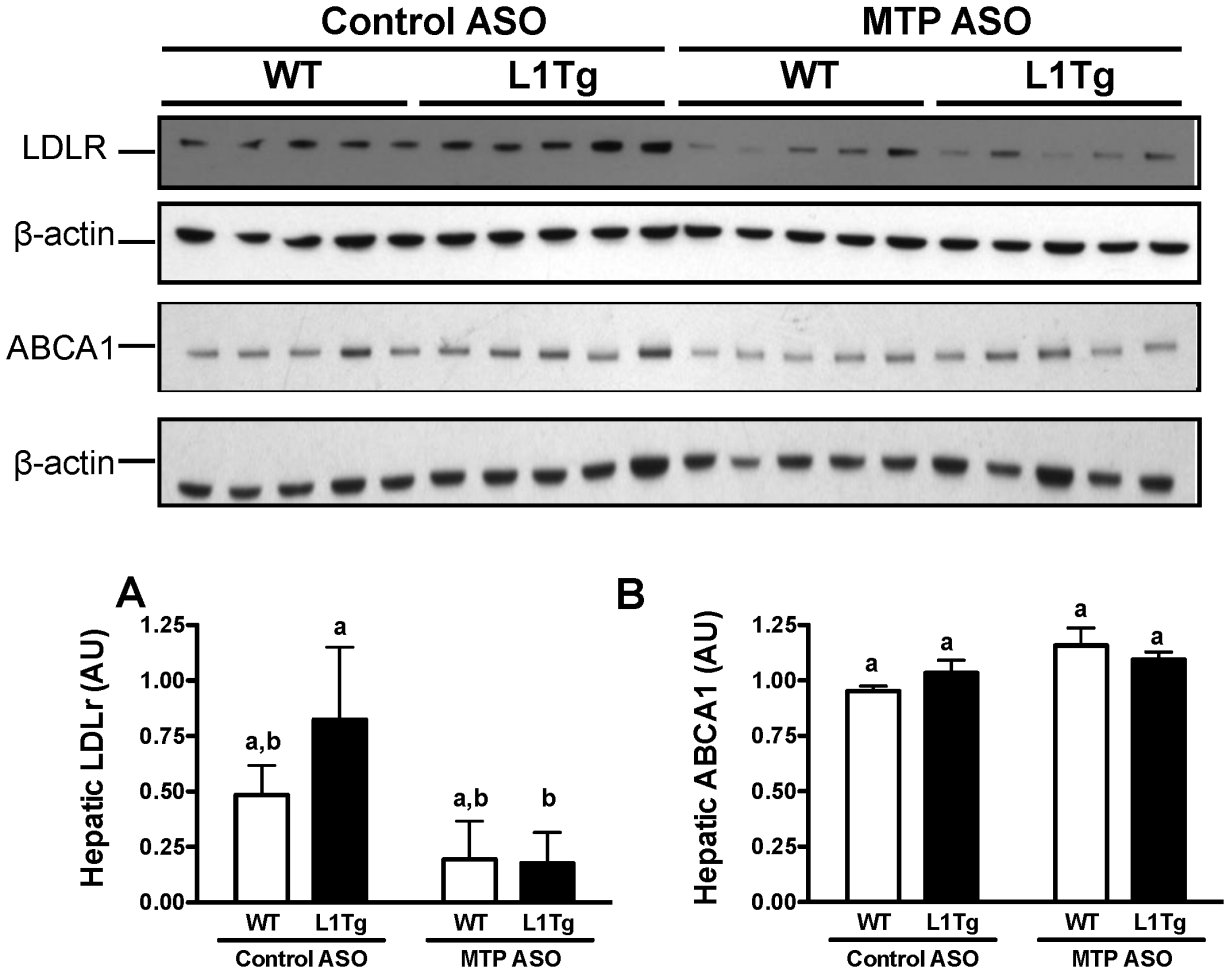
Liver expression of LDLR and ABCA1 protein in mice with hepatic MTP knockdown. Following 6 weeks of control ASO or MTP ASO treatment, liver was collected for immunoblot analysis of LDLR and ABCA1. To quantify protein expression, band intensity for LDLR (A) and ABCA1 (B) was measured by densitometry and normalized to the band intensity of β-actin. Data represent the means ± SEM (n = 4–5 mice per treatment group), and means not sharing a common superscript differ significantly (*p*<0.05).

In spite of the significant reduction in hepatic lipid secretion on apoB-containing lipoproteins (Figure 4), the plasma concentration of cholesterol and apoB associated with LDL was either increased or unchanged in WT and L1Tg mice with MTP^HKD^ (Figure 5C,5I). This finding was partially explained by the continued secretion of apoB in the face of MTP^HKD^ (Figure 4E). In addition, because of the significant increase in liver cholesterol caused by MTP ASO treatment (Figure 2A,B), it was hypothesized that hepatic LDL receptor (LDLR) was downregulated consequently resulting in reduced LDL clearance. Immunoblot analysis of liver verified that LDLR was reduced in both WT and L1Tg mice with MTP^HKD^ (Figure 6A). Since HDLc was significantly reduced in MTP ASO-treated L1Tg, hepatic ATP-binding cassette transporter (ABCA1) expression was also measured. However, there was no significant difference in liver ABCA1 protein amongst the different treatment groups (Figure 6B).

## Discussion

We hypothesized that hepatic apoB-containing lipoproteins were involved in delivering cholesterol to the small intestine for TICE. We tested this hypothesis by reducing hepatic MTP expression in WT mice and L1Tg mice, which primarily excrete cholesterol via TICE. MTP ASO treatment reduced hepatic MTP protein expression by >75% (Figure 1), increased hepatic neutral lipid content by >300% (Figure 2), and decreased VLDL TC and TG secretion by >50% (Figure 4). Although biliary cholesterol concentration was not altered, fecal cholesterol excretion was reduced by ∼50% in L1Tg treated with MTP ASO (Figure 3). From these results, we conclude that MTP-dependent lipidation of hepatic apoB-containing lipoproteins is necessary for proper maintenance of TICE. Moreover, our data are the first to indicate that VLDL or a catabolic product such as LDL is responsible for delivering cholesterol thorough the plasma to the small intestine for TICE.

When treated with MTP ASO, WT and L1Tg mice displayed similar reductions in secretion of cholesterol and TG on apoB-containing lipoproteins (Figure 4). However, MTP^HKD^ decreased fecal cholesterol excretion in L1Tg but not WT mice (Figure 3). The inability of MTP ASO treatment to reduce fecal cholesterol excretion in WT mice could be due to the hepatobiliary pathway being intact. Under normal conditions in mice, ∼70% of cholesterol excreted in feces is derived from the bile [6]–[8]. Thus, in WT mice with MTP^HKD^, liver cholesterol normally destined for TICE could have been rerouted into the bile. Alternatively, because apoB-containing lipoprotein secretion was not completely abolished, TICE could have been only partially inactivated in the WT mice with MTP^HKD^ thus resulting in a reduction in TICE-derived cholesterol excretion that was below our level of detection.

Our conclusion that hepatic apoB-containing lipoproteins support the TICE pathway is consistent with the recent results reported by Le May et al [27]. Using intestinal explants from LDLR deficient mice, it was found that LDL-derived TICE was decreased by 58%. In contrast, PCSK9 deficient mice, which displayed ∼300% increase in intestinal LDLR, had a ∼60% increase in LDL-derived TICE. These data indicate that the LDLR feeds cholesterol into TICE by internalizing apoB-containing lipoproteins at the basolateral surface of enterocytes and are in concordance with our observation of an ∼50% decrease in TICE when hepatic VLDL secretion was disrupted.

We believe that TICE was decreased in L1Tg mice with hepatic MTP knockdown due to reduced secretion of cholesterol on apoB-containing lipoproteins. However, MTP^HKD^ in L1Tg mice also resulted in a significant drop in plasma HDL cholesterol (Figure 5E) thus raising the possibility that the reduction in HDL was responsible for the diminution in TICE. Although HDL is believed to play a major role in hepatobiliary reverse cholesterol transport, data from our lab and those of others indicates that HDL does not directly participate in TICE. Intestinal uptake of HDL CE was similar in WT and ABCB4^−/−^ mice, which predominantly excrete cholesterol via the TICE pathway [14]. In addition, LXR agonist treatment, which raises TICE ∼2-fold [6], reduced HDL [3H]-cholesteryl ether accumulation in the intestine of both WT and ABCB4^−/−^ mice [14]. Because of an inability to form nascent HDL, ABCA1 deficient mice (ABCA1^−/−^) have very low levels of plasma HDL. Nevertheless, Plosch and colleagues reported that LXR agonist treatment raised fecal neutral sterol excretion to the same extent in ABCA1 deficient mice and WT littermate controls [17]. Moreover, it has been recently shown that TICE as measured by intestinal perfusion was similar in WT and ABCA1^−/−^ mice [16]. Scavenger receptor B-I (SR-BI) mediates the selective uptake of cholesterol from HDL and is expressed in the intestine [28]. Yet mice with SR-BI deficiency (SR-BI^−/−^) have been shown to have either increased [12] or unaltered TICE [16] as measured by intestinal perfusion. Intestinal uptake of [3H]-cholesteryl ether from HDL was surprisingly increased in SR-BI^−/−^ mice and was reduced when these mice were treated with LXR agonist [14]. In addition, a recent study by our group showed that overexpression of SR-BI in the intestine of WT and L1Tg mice had no effect on fecal neutral sterol excretion [29]. ApoE-rich HDL accumulate in the plasma of L1Tg suggesting that these lipoproteins could be involved in TICE [15]. However, our unpublished analysis of L1Tg mice lacking apoE indicates that the absence of apoE-rich HDL has no impact on fecal neutral sterol excretion and presumably TICE. Based upon the published data and our findings reported in this work, we believe that hepatic apoB-containing lipoproteins and not HDL are primarily responsible for feeding cholesterol into the TICE pathway.

Based upon the data presented in the current work, we propose the following mechanism for TICE in L1Tg mice. Cholesterol deposited into the liver by HDL or LDL is trafficked to the basolateral membrane of hepatocytes and pumped into the bile through the action of ATP binding cassette transporters G5 and G8 (ABCG5/G8). NPC1L1 draws the cholesterol out of the bile and similar to the small intestine directs the cholesterol to MTP and acyl-CoA cholesterol acyltransferase 2 (ACAT2) that package the cholesterol into apoB-containing lipoproteins such as VLDL. The VLDL or a catabolic product such as LDL is trafficked to the small intestine. Following internalization by the LDLR or another cell surface receptor, the cholesterol is moved across the enterocytes and is effluxed into the intestinal lumen via ABCG5/G8. Obviously, many of the steps in our hypothetical model of TICE need to be verified by additional studies. However, our finding that hepatic apoB-containing lipoproteins feed cholesterol into TICE should facilitate the discovery of the proteins that target VLDL or LDL to enterocytes and the intestinal receptors that internalize these lipoproteins.
